# Supplementary material for: Heterogeneous CaMKII-Dependent Synaptic Compensations in CA1 Pyramidal Neurons From Acute Hippocampal Slices
Source: Front Cell Neurosci. 2022 Mar 30;16:821088. doi: 10.3389/fncel.2022.821088 (PMC9005847; doi:10.3389/fncel.2022.821088)
Supplement: Supplementary file 1 [file Data_Sheet_1.PDF]

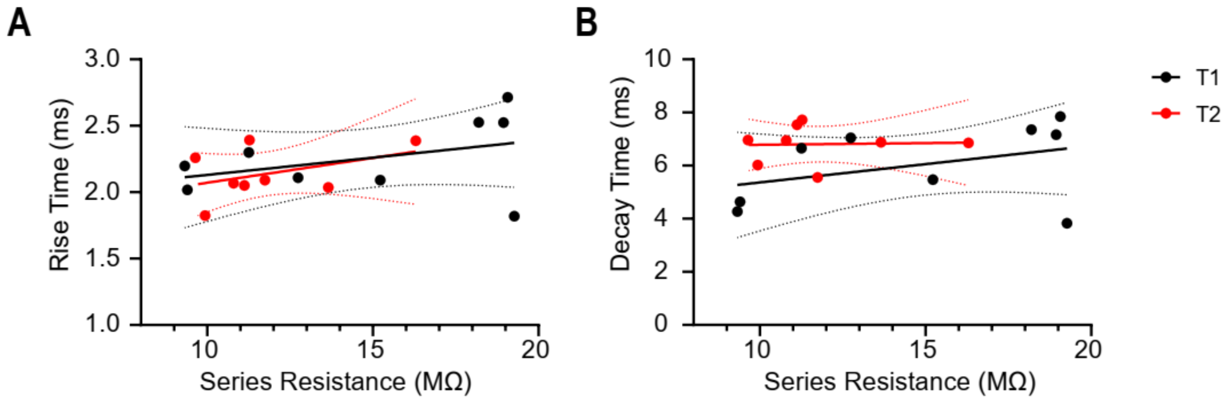

Supplementary Figure 1. No significant correlation between mEPSC average kinetics and series resistance (data from Figure 1).

(A) Correlation plot between average rise time and series resistance. Each point represents a neuron. (T1: r = 0.3831, Pearson correlation *P* = 0.3088; T2: r = 0.419, Pearson correlation *P* = 0.3004).
(B) Correlation plot between average decay time and series resistance. (T1: r = 0.3889, Pearson correlation *P* = 0.3009; T2: r = 0.038, *P* = 0.9283).

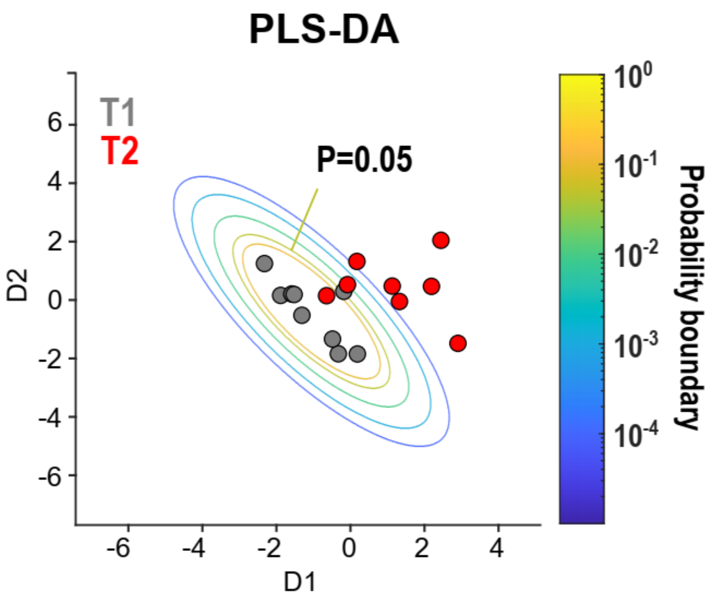

Supplementary Figure 2. Synaptic and intrinsic properties observed at T2 are unlikely to be found at T1 (data from Figure 1).

Partial least squares-discriminant analysis projection of the synaptic and electrophysiological properties (mEPSC amplitude, frequency, rise, decay, input resistance, membrane capacitance, membrane noise) of neurons recorded at T1 and T2. Error ellipses correspond to different iso-probability boundaries (0.9, 0.95, 0.99, 0.999, 0.9999) for T1 data.

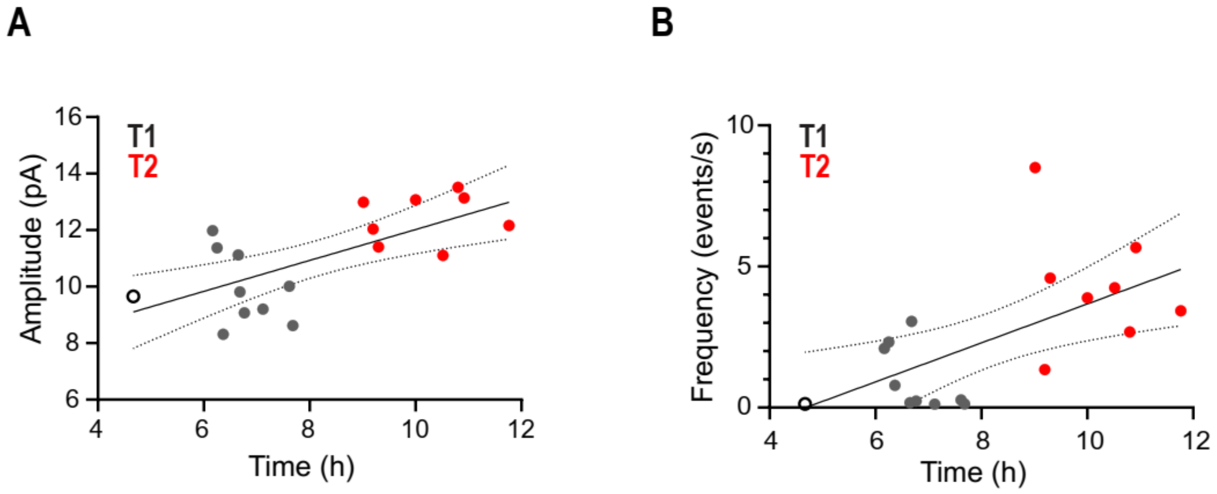

Supplementary Figure 3. Time-dependent changes in mEPSC average amplitude and frequency showing that before 5 hours event incidence is close to zero (data from Figure 1).

(A) Linear trend of average amplitude vs time (r = 0.67; Pearson correlation P = 0.002). (B) Linear trend of average frequency vs time (r = 0.60; Pearson correlation P = 0.008). Note that the linear trend of frequencies intercepts the x-axis at 4.7 h. The hollow circle corresponds to a neuron recorded before 5h.

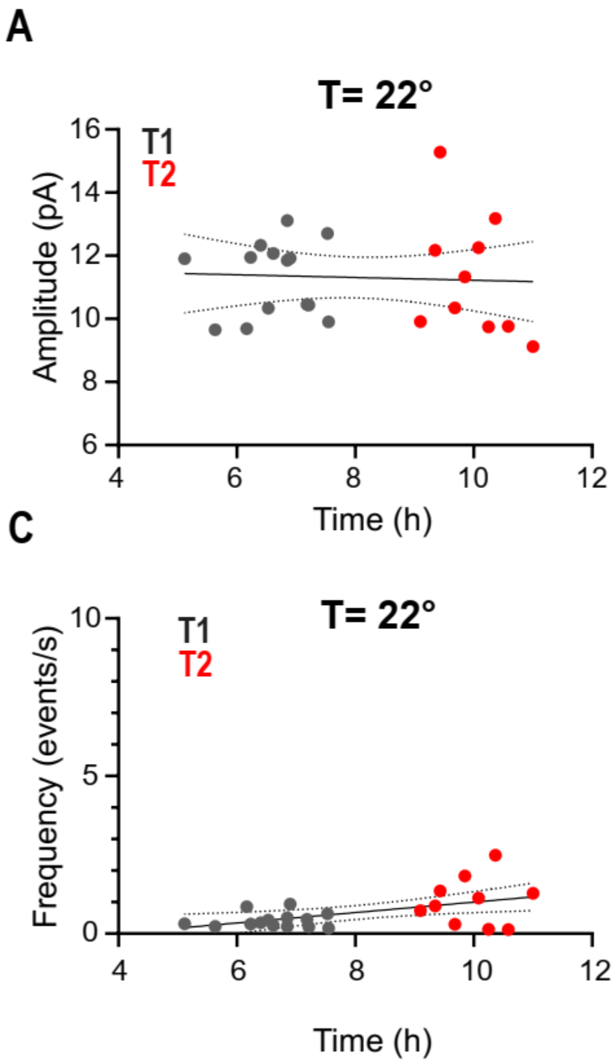

Supplementary Figure 4. Synaptic adaptations depend on preincubation temperature.

(A) Linear trend of the average amplitude vs time at 22°C (r = -0.05; Pearson correlation P = 0.809). (B) mEPSC amplitude at T1 and T2 at 22°C. Unpaired t-test: t(22) = 0.001, P = 0.999. (C) Linear trend of average frequency vs time at 22°C (r = 0.5; Pearson correlation P = 0.012). (D) mEPSC frequency at T1 and T2 at 22°C. Welch's t-test: t(10.26) = 2.43, P = 0.035.

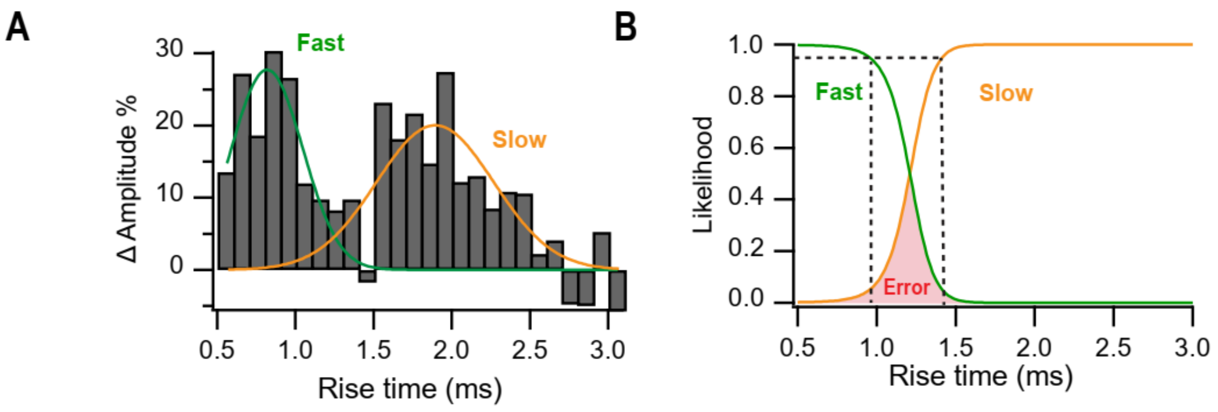

Supplementary Figure 5. Empirical classification as fast- and slow-rising populations according to their change with time (data from Figure 2).

(A) To separate fast from slow events a bimodal distribution was fitted to the %Δ Amplitude vs rise time data. The green and orange lines show the probability density function of the underlying gaussian curves constituting the fast and slow groups.
(B) These probabilities can be used to estimate the likelihood that an event corresponds to the fast or slow group.

At a given rise time τ the likelihood to belong to the fast group is:

$$L_{fast}(\tau_r) = P_{fast}(\tau_r)/[P_{fast}(\tau_r) + P_{slow}(\tau_r)]$$

And to the slow group:

$$L_{slow}(\tau_r) = 1 - L_{fast}(\tau_r)$$

For classification, only events with a maximum likelihood of 0.95 were considered. For events with rise time between 1 - 1.4 ms, the maximum likelihood was below 0.95. These events were categorized as “intermediate,” given that it is not possible to separate them into fast or slow.

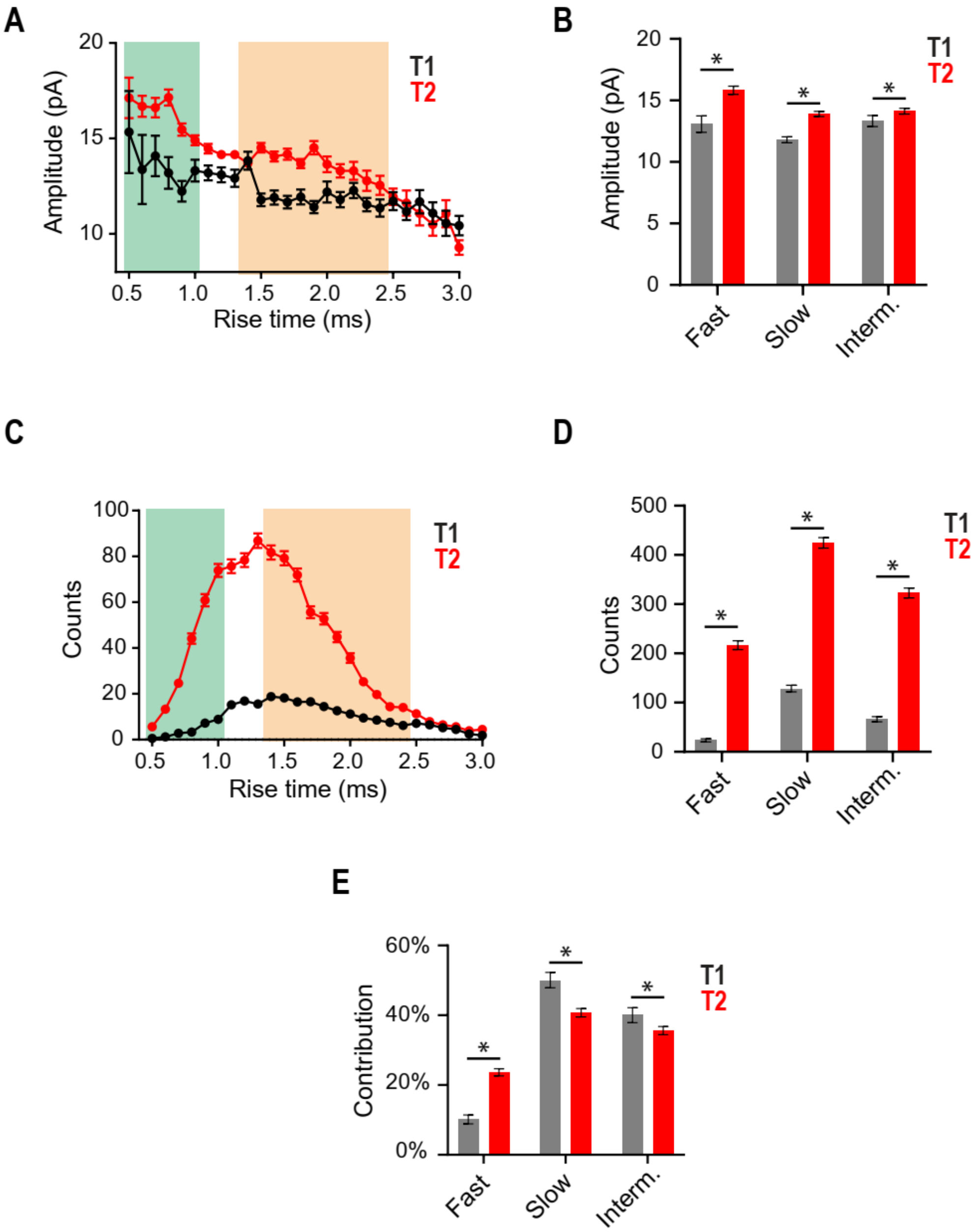

Supplementary Figure 6. mEPSC amplitude and frequency at T1 and T2 for fast, slow and intermediate events (data from Figure 2).

(A) Overlaid τ_r-sorted mEPSC amplitude distributions for T1 and T2. (B) Mean mEPSC amplitudes at T1 and T2 for fast, slow and intermediate events. (C) Overlaid τ_r-sorted mEPSC count for T1 and T2. (D) Mean mEPSC count at T1 and T2 for fast, slow, and intermediate events. (E) Contribution of fast, slow, and intermediate events to the cumulative summation of mEPSC amplitudes. The amplitudes of fast, slow, or intermediate events were summated and divided by the sum of all events. Bootstrap with Bonferroni correction. *P<0.05.

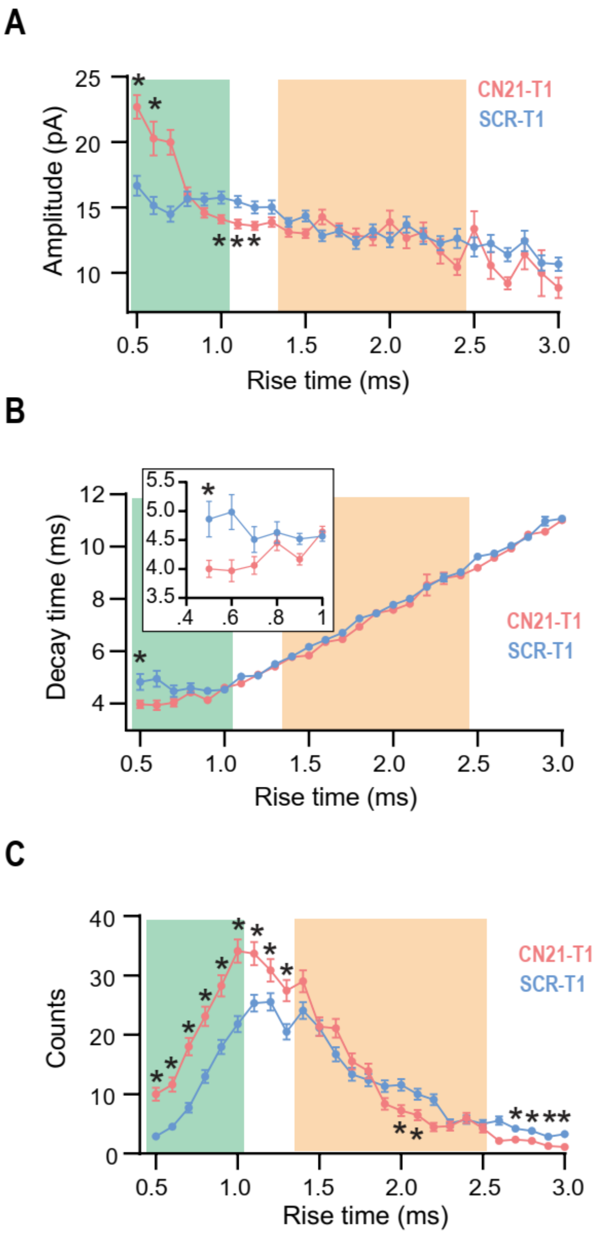

Supplementary Figure 7. Homeostatic adaptations detected at T1 upon early CaMKII inhibition (data from Figure 3).

(A) Overlaid τ_r-sorted mEPSC amplitude distributions at T1, revealing that after CN21 transmission was already increased for the faster rising group and diminished for an intermediate population. (B) Same as (A), for decay time. Inset, detail of the curves for the faster-rising subgroup, unraveling an earlier τ_r decrease after CN21 in this group. (C) Comparison of event frequency distributions at T1 also shows an incipient upregulation of faster-rising events incidence after CN21. Bootstrap with weighted P-values (all panels). *P<0.05.
