# Supplementary material for: Heterogeneous CaMKII-Dependent Synaptic Compensations in CA1 Pyramidal Neurons From Acute Hippocampal Slices
Source: Front Cell Neurosci. 2022 Mar 30;16:821088. doi: 10.3389/fncel.2022.821088 (PMC9005847; doi:10.3389/fncel.2022.821088)
Supplement: Supplementary file 2 [file Data_Sheet_2.docx]

**Supplementary Table 1**

Summary Data and Statistics for Figures 1B, E. Unpaired t-test

**Supplementary Table 2**

Summary Data and Statistics for Figure 2. Percentile bootstrap with Bonferroni correction.

**Supplementary Table 3**

Summary Data and Statistics for Figures 3C, D. Two-way ANOVA with Bonferroni correction.

**Supplementary Table 4**

Summary Data and Statistics for Figures 3F, L. Percentile bootstrap with Bonferroni correction.
